# Standardized Handwriting to Assess Bradykinesia, Micrographia and Tremor in Parkinson's Disease

**DOI:** 10.1371/journal.pone.0097614

**Published:** 2014-05-22

**Authors:** Esther J. Smits, Antti J. Tolonen, Luc Cluitmans, Mark van Gils, Bernard A. Conway, Rutger C. Zietsma, Klaus L. Leenders, Natasha M. Maurits

**Affiliations:** 1 Department of Neurology, University Medical Center Groningen, University of Groningen, Groningen, the Netherlands; 2 VTT Technical Research Centre of Finland, Tampere, Finland; 3 The Department of Biomedical Engineering, University of Strathclyde, Glasgow, United Kingdom; 4 Manus Neurodynamica Ltd, Newcastle, United Kingdom; Charité-Universitätsmedizin Berlin, Germany

## Abstract

**Objective:**

To assess whether standardized handwriting can provide quantitative measures to distinguish patients diagnosed with Parkinson's disease from age- and gender-matched healthy control participants.

**Design:**

Exploratory study. Pen tip trajectories were recorded during circle, spiral and line drawing and repeated character ‘elelelel’ and sentence writing, performed by Parkinson patients and healthy control participants. Parkinson patients were tested after overnight withdrawal of anti-Parkinsonian medication.

**Setting:**

University Medical Center Groningen, tertiary care, the Netherlands.

**Participants:**

Patients with Parkinson's disease (n = 10; mean age 69.0 years; 6 male) and healthy controls (n = 10; mean age 68.1 years; 6 male).

**Interventions:**

Not applicable.

**Main Outcome Measures:**

Movement time and velocity to detect bradykinesia and the size of writing to detect micrographia. A rest recording to investigate the presence of a rest-tremor, by frequency analysis.

**Results:**

Mean disease duration in the Parkinson group was 4.4 years and the patients were in modified Hoehn-Yahr stages 1–2.5. In general, Parkinson patients were slower than healthy control participants. Median time per repetition, median velocity and median acceleration of the sentence task and median velocity of the elel task differed significantly between Parkinson patients and healthy control participants (all p<0.0014). Parkinson patients also wrote smaller than healthy control participants and the width of the ‘e’ in the elel task was significantly smaller in Parkinson patients compared to healthy control participants (p<0.0014). A rest-tremor was detected in the three patients who were clinically assessed as having rest-tremor.

**Conclusions:**

This study shows that standardized handwriting can provide objective measures for bradykinesia, tremor and micrographia to distinguish Parkinson patients from healthy control participants.

## Introduction

Parkinson's disease (PD) is a neurodegenerative disorder which generally results in several motor symptoms. The cardinal signs of the disease are bradykinesia (slowness of movement), rest tremor, rigidity (muscular stiffness throughout the range of passive movement in a limb segment) and postural and gait impairment [1]. Not all PD patients present these classical symptoms and several other motor symptoms can be observed, such as freezing, shuffling gate, hypomimia and micrographia (small handwriting) [2]. Clinical examination can be expressed in rating scales, e.g. the Unified Parkinson's Disease Rating Scale (UPDRS) or the Hoehn and Yahr scale (H&Y) [2], [3]. The UPDRS is the most widely used and tested scale and consists of an impairment and disability section. The H&Y scale is the most commonly used method to assess the severity of the disease [4]. However, rating scales highly depend on the experience and interpretation of the physician performing the assessment and have limited precision for quantifying upper limb motor skill. To support the clinical diagnosis, a trial dose of levodopa should result in an improvement of the clinical symptoms. The clinical diagnosis can also be supported by radiotracer neuroimaging techniques such as positron emission tomography or single photon emission computed tomography, in which a presynaptic dopaminergic deficit can be demonstrated [5].

Early diagnosis of PD is very important, because it allows early intervention and management toward an improved overall outcome for the patient [6]. Currently, no definite methods for an early, objective and quantitative diagnosis are available, but several methods that provide quantitative measures for motor symptoms of PD have been studied. For example, handwriting tasks and systems have been used for this purpose [7]–[13]. Bajaj et al. [7] used handwritten samples to differentiate PD patients from patients with other tremors. They provided an objective measure for micrographia, but their analysis was time consuming, because script height and length were measured manually. An electronic pen and digitizer tablet were used in other studies to distinguish PD from healthy control (HC) participants [8], [10], [12]. However, Alty et al. [8] only studied bradykinesia and Van Gemmert et al. [12] only studied micrographia. Broderick et al. [10] studied both micrographia and bradykinesia, but the shoulder and elbow of participants were fixated, which resulted in a constrained, rather unnatural movement. Ünlü et al. [9] used an electronic pen as well and showed that several features can be computed to distinguish PD from HC. One of the features was related to tremor, but the remaining features were not related to a symptom of PD. Rosenblum et al.[13] also used handwriting to distinguish PD from HC analyzing movement speed and size of writing. They did not assess tremor. Thus, each of the systems provided useful measures to distinguish PD from HC participants, but most of them focused on just one of the motor symptoms of PD and none of these studies included a task to measure rest tremor. For early differential diagnosis a system which provides quantitative measures for several motor symptoms of PD simultaneously would be beneficial [14].

The aim of the present study was to determine whether standardized handwriting can provide quantitative measures to assess multiple important motor symptoms simultaneously to distinguish patients diagnosed with PD from age- and gender matched HC participants. The study focused on two important motor symptoms of PD, bradykinesia and micrographia. Additionally, rest tremor was investigated. The design of the present study was exploratory and therefore a small group of PD patients and HC participants was included and a large number of features was produced, to examine which features can best be used to distinguish PD from HC.

Several handwriting and geometric tasks, based on tasks used in previous studies, were evaluated. Ünlü et al. [9] used the writing of l-loops and a complete sentence. In a study of Ponsen et al.[15] participants wrote a complete sentence and the authors showed that letter height decreased in PD patients as writing progressed. Also Bajaj et al.[7] assessed micrographia in PD by analyzing a handwritten sentence. The present study includes the writing of e- and l-loops and a complete sentence to assess micrographia.

Besides writing tasks, geometric tracing tasks were included in this study, based on previous findings. For example, Keresztényi et al.[11] used a circle tracing task to show that PD patients were significantly slower than HC. Other studies [16], [17] also investigated a circle drawing task to compare PD with HC. Saunders-Pullman et al.[18] showed a correlation between spiral analysis and the UPDRS score and Stanley et al.[19] described that spiral analysis may be more sensitive than the UPDRS for detecting early changes in motor performance. Dounskaia et al.[16] showed that drawing lines in different directions differentiated between PD and HC. For example, line drawing variability was higher in PD than in HC. Therefore, in the present study line drawing in eight different directions was included in addition to circle and spiral tracing tasks. A rest task was added as well, based on the task used by Scanlon et al.[20] to measure rest tremor.

To summarize, the present study aimed to provide quantitative measures to evaluate bradykinesia, micrographia and tremor in one assessment by recording pen tip movement during handwriting tasks, including tracing geometric figures and actual writing. We additionally assessed whether these features allowed distinguishing PD patients from HC participants.

## Methods

### Ethics Statement

The study protocol was approved by the Medical Ethical Committee of the University Medical Center Groningen.

### Participants

Ten patients with PD (mean age 69.0 years; range 63–81, 6 male) and ten gender- and age- matched HC participants (mean age 68.1 years; range 61–78, 6 male) participated. Patients, who are clinically diagnosed with PD by a neurologist (according to the United Kingdom Parkinson's Disease Society Brain Bank Diagnostic Criteria for Parkinson's Disease [21]) and who are under treatment at the movement disorders clinic in the University Medical Center Groningen (UMCG) were contacted retrospectively. Since the patients had to be able to hold a pen for 30 minutes and perform tracing and writing tasks, PD patients in relatively early stages of the disease (modified H&Y stage 1–2.5 [3], [22]) were selected. The first ten patients who replied positively and met the inclusion criteria were included. The healthy participants were recruited from the general population and were matched to the patients by their age and gender. All participants were right-handed according to the Annett handedness scale [23] and signed informed consent before participation. All PD patients complied with overnight withdrawal of PD-related medication. Exclusion criteria were a history of epileptic seizures, head injury, neurological disorders (other than PD for the patients), the use of medication affecting movement, or a low (<26) score on the Mini Mental State Examination (MMSE). Patients who suffered from a severe tremor in the hands (score ≥3 on the UPDRS-III) were excluded from the study, because this study mainly focused on bradykinesia and micrographia. Table 1 shows a summary of the patient characteristics.

**Table 1:** PD patient characteristics.

Patient no.	Age (years)	Gender	Disease duration (years)	Modified Hoehn and Yahr scale	UPDRS score (last visit)
1	63	M	5	2	6
2	81	M	5	2.5	35
3	79	M	3	2	20
4	78	M	4	2	18
5	62	F	4	1	11
6	64	M	4	1.5	12
7	67	F	8	1.5	13
8	67	M	5	1	*
9	65	F	2	1.5	11
10	64	F	4	1.5	11

*No UPDRS score was available for this patient.

### Experimental design

Participants were seated in front of a table in a comfortable position to write. As was shown before [9], [14], [16], a digitizer pen and tablet are suitable to record handwriting. A graphic tablet^a^ (WACOM Intuos 2) and a modified digitizer pen were used. The position of the pen-tip on the tablet during movement was recorded using the MovAlyzeR software^b^ (Neuroscript LLC, USA) with a sampling frequency of 100 Hz. The pen had a wired connection to an operator computer where MovAlyzeR was installed. Participants performed five drawing and writing tasks (see below) using the digitizer pen. The examiner was seated behind the operator computer and determined whether the participants executed the tasks correctly. If a task was executed incorrectly, the recording was stopped and restarted after re-instruction. An example of incorrect task execution would be moving the pen in the wrong direction or starting the task too early.

### Tasks

**Figure 1:**
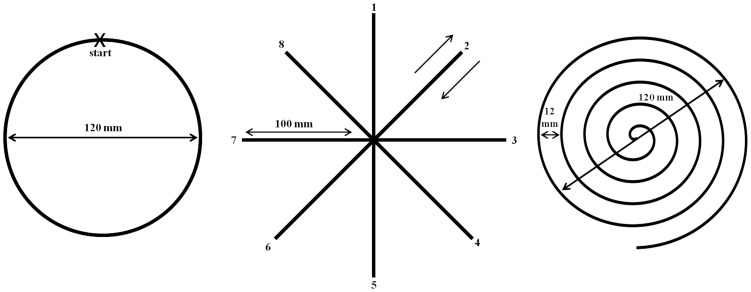
Templates used for tracing geometric shapes; circle, star and spiral. The dimensions of the templates are indicated in the figure.

Each participant performed five tasks in the same order, to limit variability in task results. Participants were instructed to start the task at a signal of the examiner and to perform the tasks at a comfortable speed, allowing them to write and draw as smoothly as individually possible. First, a rest recording (30 seconds) was performed prior to the writing and drawing tasks to measure pen movement at rest. The participants were instructed to touch the tablet with the pen-tip, with the lower right arm resting on the table [20]. Next, the participants traced geometric shapes on templates; a circle, a star and a spiral (Figure 1). The templates were printed on A4 paper and placed on the tablet under a transparent sheet.

#### Circle drawing

In this task, participants had to continuously trace a circle ten times in a clockwise direction starting from the 12 o'clock position (Figure 1).

#### Star drawing

Straight lines orientated in eight different directions, set at 45 degrees to each other and forming an eight pointed star, were traced in this task (Figure 1). The lines had to be repeatedly traced from the central point of the star to each endpoint and back, ten times without interruption, starting with the upward direction and then proceeding clockwise.

#### Spiral drawing

In this task the participants traced a spiral (Figure 1) clockwise from inside to outside. Each participant performed ten consecutive spiral tracing trials.

During the last two tasks, the participants wrote a particular phrase ten times. The texts were chosen such that symbols and words were written repetitively and texts were nonsensical in one case and meaningful in the other.

#### ‘elel’ character writing

In this task the participant wrote the 8 character text sequence ‘*elelelel*’ ten times with each phrase starting at the left side of the tablet.

#### Sentence writing

In this task, the participant wrote the sentence: ‘veel te veel felle schelle zon’ (‘way too much bright, shrill sun’ in Dutch), ten times.

### Data analysis

Using custom made scripts in Matlab 7.4.0 (R2007a) the drawing and writing tasks were analyzed to evaluate the speed of movement to assess bradykinesia and the size of writing to assess micrographia. Additionally, a frequency analysis was performed to assess rest-tremor. The data were preprocessed to allow for evaluation of each separate trial as well as of the whole task (see methods S1 and figure S1). The pen position data for the star task were divided into four main directions, for comparison. Directions 1 and 5 (see Figure 1) were taken together as the vertical direction, 3 and 7 as the horizontal direction, 2 and 6 as diagonal1 and 4 and as diagonal2. The data points were assigned to the main directions (see methods S1 and figure S2). Separating each line of the ‘*elel*’ task and recognizing the individual letters was done using a state vector machine (see methods S1 and figure S3). The start and end points of each sentence were selected manually.

#### Bradykinesia assessments

To assess bradykinesia, features concerning movement speed were defined. Total movement time was calculated for the circle, spiral and star task. Median time for each trial was calculated for the circle, spiral and sentence task. Median velocity and acceleration were calculated for all tasks. For the star task median time for each line was calculated for the whole task as well as for the four main directions. Finally, for the ‘*elel*’ task median times for writing an ‘e’ or an ‘l’ were calculated yielding 23 bradykinesia features in total.

#### Micrographia assessment

To assess micrographia, writing size was investigated. For the ‘*elel*’ task median width and height of the individual letters ‘*e*’ and ‘*l*’ were calculated. For the sentence task median script height and median sentence length were calculated, yielding six micrographia features in total.

### Tremor assessment

Data collected during the rest task were used to investigate the presence of a rest-tremor. To detect the tremor, the data of the pen tip location (*x* and *y*) were analyzed. First, the difference signals, *dx* and *dy*, for *x* and *y* were computed according to:
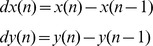
where *n* is the signal's sample index. Then a principal component analysis was performed and the first principal component of *dx* and *dy* was selected, to take into account all possible directions for rest-tremor. The first principal component is the linear combination of *dx* and *dy* with the highest variance. The power spectrum of the first principal component was computed using Welch's method. Finally, the spectral maximum was identified and the power spectral density (PSD) and frequency at the peak were determined.

### Statistical analysis

Statistical analyses were conducted using SPSS 20.0.0.1. First, it was tested whether features were normally distributed by the Shapiro-Wilk test. For both groups, all features were described by their mean and standard deviation when normally distributed, or median and interquartile range (iqr), when not normally distributed. Since the goal was to derive quantitative measures for bradykinesia, micrographia and rest tremor and to assess whether these features could be used to distinguish PD patients from HC participants, the bradykinesia and micrographia features were compared between the two groups. Since only a few patients had rest tremor, related features were not compared further. To compare the bradykinesia and micrographia features between the two groups, multiple independent t-tests were performed for the features which were normally distributed and the Mann Whitney test was used when normality assumptions were violated. The statistical analyses were corrected for multiple comparisons by applying a Bonferroni correction. After Bonferroni correction a probability value (p) of ≤0.0014 (0.05/35) was considered significant for the bradykinesia and micrographia assessments. Additionally, to investigate the progressive reduction in writing size the difference between the first and last trial was computed for the width and height of the letters ‘e’ and ‘l’ and the length and height of the sentence and also compared between the two groups with multiple independent t-tests. After Bonferroni correction a probability value (p) of ≤0.0014 (0.05/35) was considered significant. Median time per line, which was normally distributed over participants, was compared between the four main directions of the star task according to a repeated measures ANOVA with between-subjects factor Group (PD and HC) and within-subject factor Direction (four main directions).

## Results

All participants completed each of the writing and drawing tasks. Median disease duration of the PD patients was 4.4 years (range 2–8) and nine PD patients normally used Parkinsonian medication.

### Bradykinesia assessments

Table 2 provides the test statistics for the bradykinesia features. Four bradykinesia features (median time per repetition, median velocity and median acceleration of the sentence and median velocity of the ‘elel’ task) differed significantly between PD and HC (all p≤0.0014). The remaining features also showed large differences between the two groups, although significance did not survive correction for multiple comparisons. Median time per line differed significantly between the four main directions of the star (F(3,16) = 9.35, p = 0.001), because median time per line was significantly higher in diagonal2 (0.81 s.) compared to diagonal1 (0.71 s.). No significant interaction was found.

**Table 2:** Summary of test statistics of the bradykinesia and micrographia features, mean (SD) values for both groups are provided in case of a normal distribution, otherwise Median (iqr) values are shown; for the normal distributed features an independent t-test was performed, otherwise a Mann Whitney U test was performed.

Task	Feature	PD	HC	t-value #	p-value	
Circle	Total Movement time (s)	37.27 (13.08)	22.87 (5.99)	3.17	0,0077	
Circle	Median time per repetition (s)	3.24 (2.26)°	2.19 (0.59)°	18 #	0,0150	
Circle	Median velocity (m/s)	0.11 (0.05)	0.18 (0.06)	−2.94	0,0087	
Circle	Median Acceleration (m/s^2^)	0.29 (0.32)°	0.47 (0.40)°	19 #	0,0190	
Cross	Total Movement time (s)	175.76 (66.55)	106.38 (36.52)	2.89	0,0098	
Cross	Median time per line (all) (s)	0.94 (0.34)	0.56 (0.21)	3.04	0,0070	
Cross	Median time per line (diagonal 1) (s)	0.89 (0.29)	0.54 (0.20)	3.09	0,0067	
Cross	Median time per line (diagonal 2) (s)	1.03 (0.43)	0.58 (0.25)	2.82	0,0112	
Cross	Median time per line (horizontal) (s)	0.91 (0.29)	0.62 (0.22)	2.58	0,0187	
Cross	Median time per line (vertical) (s)	0.98 (0.40)	0.53 (0.20)	3.20	0,0072	
Cross	Median Velocity (m/s)	0.11 (0.06)	0.17 (0.06)	−2.48	0,0234	
Cross	Median Acceleration (m/s^2^)	0.41 (0.44)°	0.96 (1.44)°	22 #	0,0350	
Spiral	Total Movement time (s)	122.69 (59.00)°	83.39 (40) °	14 #	0,0050	
Spiral	Median time per repetition (s)	10.36 (5.36)°	6.79 (3.79)°	16 #	0,0090	
Spiral	Median velocity (m/s)	0.10 (0.05)	0.15 (0.05)	−2.40	0,0274	
Spiral	Median Acceleration (m/s2)	0.29 (0.27)°	0.54 (0.79)°	19 #	0,0190	
Sentence	Median time per repetition	16.30 (4.94)°	11.18 (2.92)°	3 #	0,0000	*
Sentence	Median velocity (m/s)	0.05 (0.02)	0.08 (0.02)	−4.22	0,0005	*
Sentence	Median Acceleration (m/s^2^)	0.78 (0.32)	1.77 (0.39)	−6.23	0,0000	*
Elel	Median velocity (m/s)	0.07 (0.04)	0.15 (0.04)	−4.18	0,0006	*
Elel	Median Acceleration (m/s^2^)	0.61 (0.36)	1.52 (0.71)	−3.62	0,0030	
Elel	Median duration letter e (s)	0.48 (0.13)	0.37 (0.10)	2.17	0,0441	
Elel	Median duration letter l (s)	0.74 (0.26)	0.51 (0.14)	2.53	0,0209	
	**Micrographia features**					
Elel	Median Width of the e (mm)	7.67 (3.42)	14.16 (3.34)	−4.29	0,0004	*
Elel	Median Height of the e (mm)	16.30 (6.57)	24.29 (5.90)	−2.86	0,0104	
Elel	Median Width of the l (mm)	12.82 (4.83)	19.15 (5.40)	−2.76	0,0129	
Elel	Median Height of the l (mm)	42.64 (15.45)	59.86 (13.82)	−2.63	0,0171	
Sentence	Median Script Height (mm)	13.46 (5.91)	18.03 (4.41)	−1.96	0,0660	
Sentence	Median Sentence Length (mm)	228.36 (116.19)°	275.96 (30.20)°	25 #	0,0630	
Elel	Difference first-last trial Width e (mm)	0.0030 (0.36)	0.13 (0.28)	−0,89	0,3870	
Elel	Difference first-last trial Height e (mm)	−0.032 (0.44)	0.37 (0.38)	−2,21	0,0410	
Elel	Difference first-last trial Width l (mm)	−0.12 (0.69)	0.25 (0.38)	−1,48	0,1560	
Elel	Difference first-last trial Height l (mm)	−0.89 (1.48)	−0.13 (0.71)	−1,46	0,1620	
Sentence	Difference first-last trial Script Height (mm)	−2881.50 (2387.90)	−898.60 (2138.36)	−1,96	0,0660	
Sentence	Difference first-last trial Script Length (mm)	−271.00 (297.80)	48.50 (410.81)	−1,99	0,0620	

°Median (iqr).

SD = Standard Deviation.

iqr = interquartile range.

# The values which are marked with a # are the U-values of the Mann Whitney U test, otherwise a t-value is shown.

*indicates a Bonferroni corrected significant result at α = 0.0014.

### Micrographia assessments

**Figure 2:**
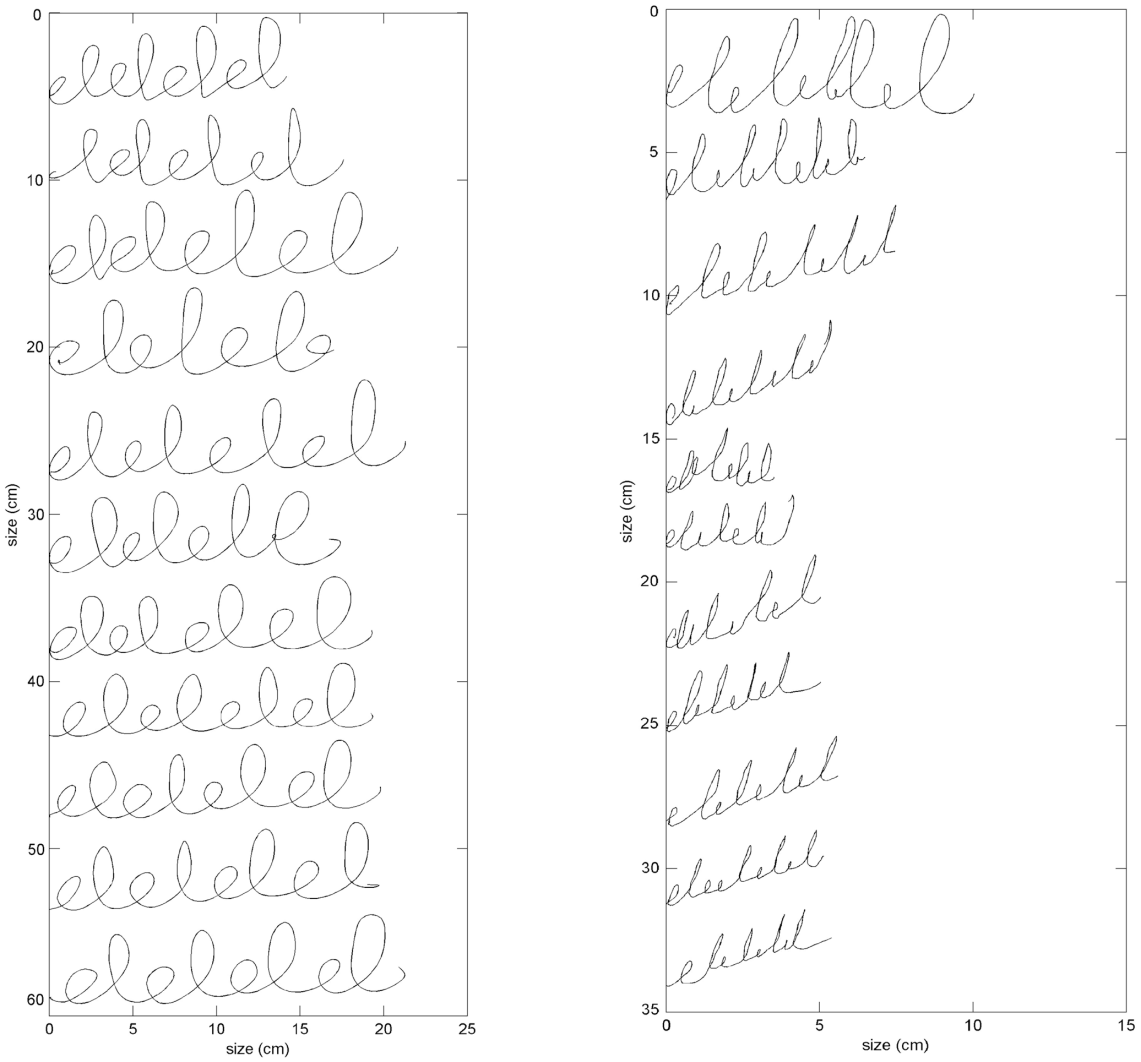
An example of the ‘*elel*’ task is shown for a HC participant (left) and a PD patient (right). Each line of the writing task was shifted vertically so that individual trials are visible. Note the differences in the x-axis and y-axis between the left and right figure.

The test statistics for the micrographia assessments are also shown in Table 2. Sentence length and sentence script height did not differ significantly between PD and HC. The width of the letter ‘e’ was significantly smaller in PD than in HC (p≤0.0014). The height of the letter ‘e’ and the width and height of the letter ‘l’ in the ‘elel’ task were smaller in PD compared to HC, although significance did not survive correction for multiple comparisons (see Figure 2 for an example of writing). No other significant effects were found concerning writing size and progressive reduction in writing size.

### Tremor assessments

The PSD at the peak was higher (>30 (mm/s^2^)/Hz) for three PD patients who were clinically assessed as having rest-tremor, than for all other participants (<2 (mm/s^2^)/Hz). The peak frequencies for these patients were between 4.4 and 8 Hz (PD2 8.0 Hz; PD3 5.3 Hz; PD7 4.4 Hz).

## Discussion

The present study showed that handwriting tasks can provide objective measures for bradykinesia, micrographia and rest tremor that distinguish PD from HC.

Corresponding to earlier studies [8], [10], [11], [13], [15] results from the current study showed that PD patients perform movements significantly slower than HC. PD patients were likely slower than HC, because of bradykinesia [10]. However, some caution is needed when drawing this conclusion, because it is crucial to distinguish bradykinesia from simple age-related slowness [1]. However, the groups were age-matched, which suggests that the decreased movement speed in PD patients reflects bradykinesia rather than just age-related slowness. All bradykinesia features showed large differences between the two groups, but only four features were significantly different between the two groups. These four features were derived from data obtained during the writing tasks, which were more complex than the tracing tasks. Moroney et al. [24] also showed in a simulation model that PD patients were slower than HC in both simple and complex movements, but slowness increased with increased movement complexity.

Writing size was examined to find objective measures for micrographia. Micrographia is a symptom frequently associated with PD and is reflected in smaller sized writing patterns [25], [26], but has also been defined as a progressive reduction in amplitude during a writing task [26]. In the current study PD patients produced smaller handwriting than HC as represented by smaller average width and height of the letters ‘e’ and ‘l’ in the ‘*elel*’ task (note that only the width of the letter ‘e’ differed significantly between groups). This result was similar to the findings of Van Gemmert et al.[12] and Rosenblum et al.[13]. They showed reduced stroke sizes in PD patients compared to HC participants who performed handwriting tasks. We investigated the progressive reduction in writing size during a task as well, and there was a small reduction in size of different letter features, but there were no significant differences between the two groups. This result is in contrast with observations by Ponsen et al.[15], who showed a progressive reduction in writing size in PD patients. The fact that the present study showed no progressive reduction in writing size during the tasks might be due to the lack of visual feedback on the tablet during the tasks as the stylus is non-inking. De Jong et al.[27] described that PD patients drew larger when no visual feedback was available. Ondo et al.[28] also showed that withdrawal of visual feedback during actual writing improved micrographia in PD patients. Therefore, in future studies handwriting with visual feedback should be analyzed, because this might improve the sensitivity of micrographia measures.

In addition, participants were asked how frequently they practiced handwriting in their daily lives to investigate whether the differences between groups are not a result of a lack of practice. The participants, both PD and HC, only wrote small amounts in their daily lives, such as a shopping-list, so we assume that the differences between the two groups in this study are a result of PD rather than a lack of practice.

Furthermore, rest tremor was detected in the patients who were clinically assessed as having rest tremor by the handwriting system described in this paper. The strength of combining handwriting tasks as was done in the present study is that three important motor symptoms of PD are assessed simultaneously. Handwriting tasks could be useful for screening PD in patients with mild symptoms: they are easily applicable in the clinic, since only a digitizer pen and tablet are needed to perform the measurements. Before such a handwriting system would be implemented in the clinic a future longitudinal study should investigate which participants with a high risk to develop PD, based on the handwriting measurements, will actually develop PD. Furthermore, future studies should investigate whether PD can be distinguished from other movement disorders using these handwriting tasks. Additionally, the custom made Matlab-scripts should be converted to automatic methods, which generate simple outcome measures for the clinician. Finally, handwriting analysis could also be useful for monitoring the effects of rehabilitation programs or other interventions.

One of the limitations of this study was the small sample size, which limits the number of statistically significant results. However, almost all features showed a clear difference between the groups (p<0.05), although they did not all survive Bonferroni correction. In addition, this study does not include a comparison with the clinical examination of the motor symptoms of PD. However, previous studies have already demonstrated correlations between separate handwriting tasks and clinical examinations [8], [18]. The handwriting test battery presented in this study might be further improved by including a measure for rigidity, which is one of the classical symptoms of PD [1]. However, rigidity is a symptom which is very hard to quantify, because it refers to an increased muscle tone noticed during subjective assessment by a physician during passive movements of, for example, an affected arm [1].

## Conclusions

In the present study we showed that standardized handwriting tasks can provide quantitative measures for the assessment of bradykinesia, micrographia and tremor. Several of these measures distinguished clinically diagnosed PD from HC.

## Supporting Information

Figure S1 **Illustration of the start and end areas in the circle and spiral task.** A: Circle task; B: Spiral task. Green: start area; Red: end area.
(TIF)

Figure S2 **Illustration of the star task segmentation method.** A: the original *x* (blue) and *y* (green) coordinate time series recorded by the digitizer. B: the *x* and *y* coordinates as a function of the distance travelled by the pen tip. The function fitted to the coordinates is shown in red for two points, one of which is a point where the subject has drawn an acute angle and the other is slightly after such a point. C: turning angle estimated from parameters of the functions fitted to the coordinate series (green) and fitting error of the functions (blue); the local minima of the fitting error are shown as red circles in the angle series. D: the turning points detected by the algorithm are marked in the original time series by red squares.
(TIF)

Figure S3 **Two samples of the ‘e’ and ‘l’ in the elel task.** Left: A sample of text containing one ‘e’ and one ‘l’, including the recognized characteristic points (red dots). The numbered black arrows show the states of the state vector machine. Right: An example of a real detected letter ‘e’. The light blue box indicates detected letters ‘e’. The line color indicates the state of the algorithm; black: state 1, dark blue: state 2, light green/cyan: state 3, green: state 4, red: state 0/error. Markers indicate state changes; blue upward arrow indicates transition from state 1 to 2, blue leftward arrow indicates transition from state 2 to 3, blue downward arrow indicates transition from state 3 to 4, a green circle indicates a transition from state 4 to state 1 and a red cross indicates a transition from any state to state 0 (the points were an error is recognized).
(TIF)

Methods S1 **Segmentation of the Digitizer data.**
(DOCX)
