# Supplementary figures and images for: Standardized Handwriting to Assess Bradykinesia, Micrographia and Tremor in Parkinson's Disease
Source: PLoS One. 2014 May 22;9(5):e97614. doi: 10.1371/journal.pone.0097614 (PMC4031150; doi:10.1371/journal.pone.0097614)

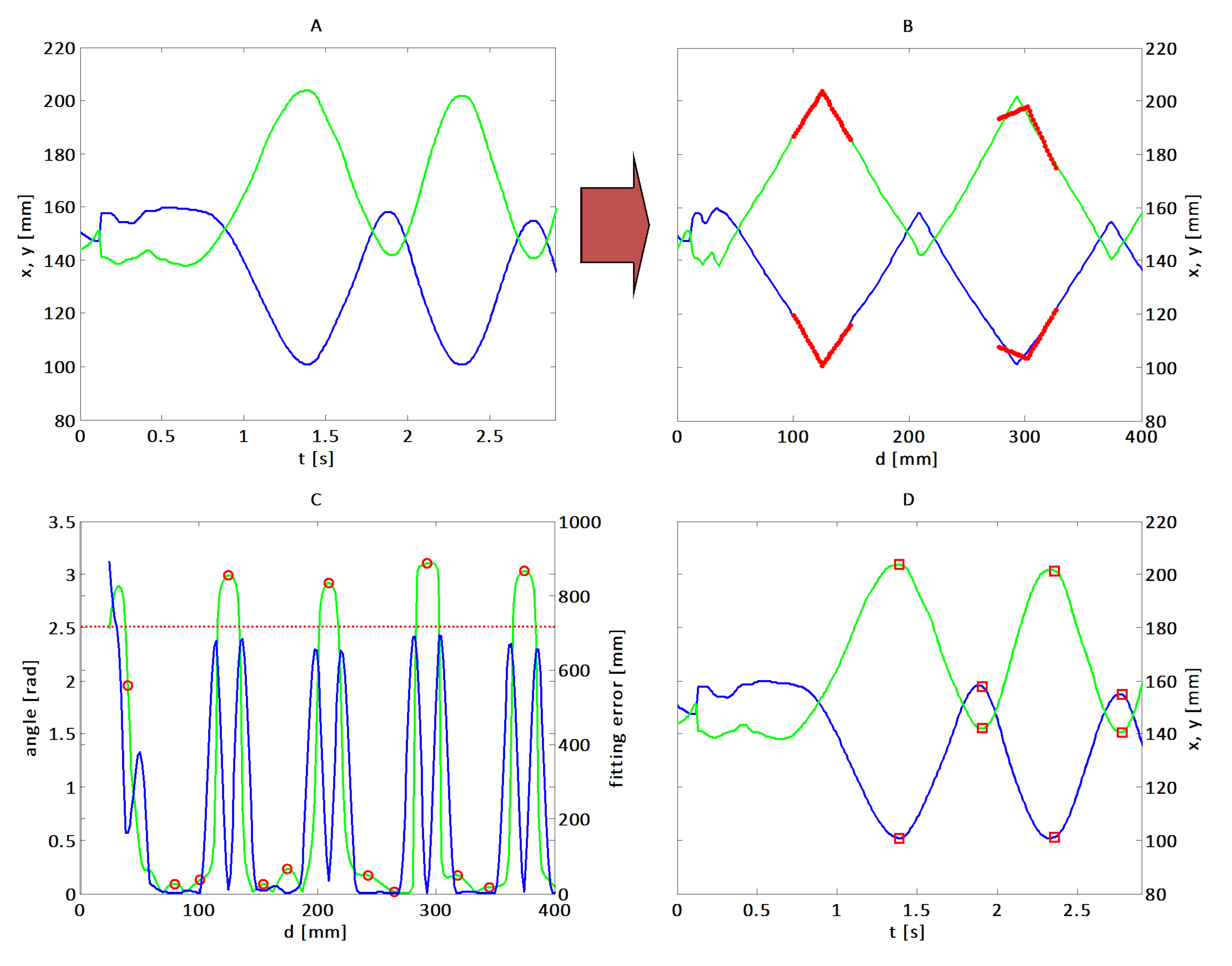

Supplement: Figure S2 — Illustration of the star task segmentation method. A: the original x (blue) and y (green) coordinate time series recorded by the digitizer. B: the x and y coordinates as a function of the distance travelled by the pen tip. The function fitted to the coordinates is shown in red for two points, one of which is a point where the subject has drawn an acute angle and the other is slightly after such a point. C: turning angle estimated from parameters of the functions fitted to the coordinate series (green) and fitting error of the functions (blue); the local minima of the fitting error are shown as red circles in the angle series. D: the turning points detected by the algorithm are marked in the original time series by red squares. (TIF) [file pone.0097614.s002.tif]

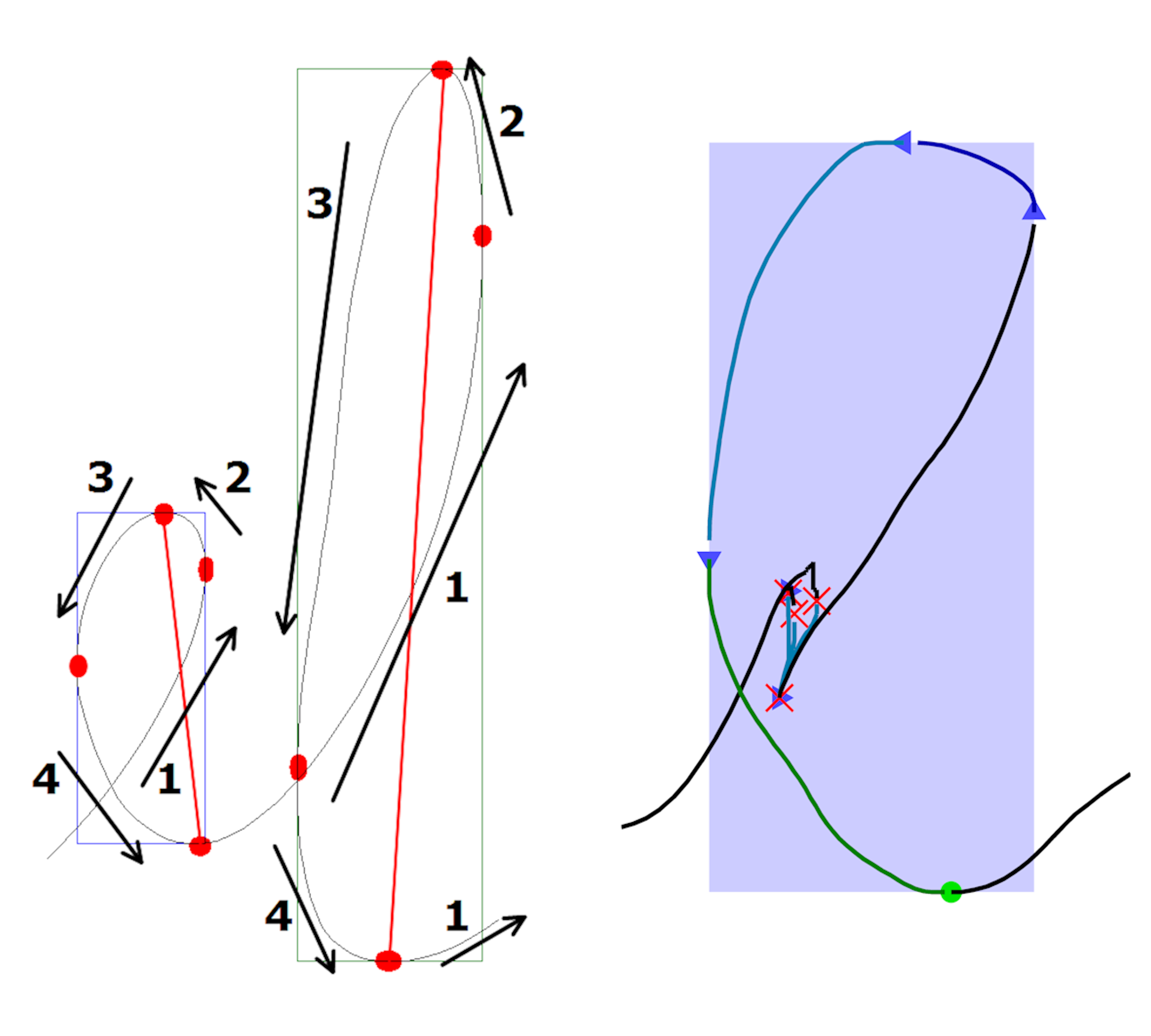

Supplement: Figure S3 — Two samples of the ‘e’ and ‘l’ in the elel task. Left: A sample of text containing one ‘e’ and one ‘l’, including the recognized characteristic points (red dots). The numbered black arrows show the states of the state vector machine. Right: An example of a real detected letter ‘e’. The light blue box indicates detected letters ‘e’. The line color indicates the state of the algorithm; black: state 1, dark blue: state 2, light green/cyan: state 3, green: state 4, red: state 0/error. Markers indicate state changes; blue upward arrow indicates transition from state 1 to 2, blue leftward arrow indicates transition from state 2 to 3, blue downward arrow indicates transition from state 3 to 4, a green circle indicates a transition from state 4 to state 1 and a red cross indicates a transition from any state to state 0 (the points were an error is recognized). (TIF) [file pone.0097614.s003.tif]
