# Supplementary material for: Standardized Handwriting to Assess Bradykinesia, Micrographia and Tremor in Parkinson's Disease
Source: PLoS One. 2014 May 22;9(5):e97614. doi: 10.1371/journal.pone.0097614 (PMC4031150; doi:10.1371/journal.pone.0097614)
Supplement: Methods S1 — Segmentation of the Digitizer data. (DOCX) [file pone.0097614.s004.docx]

**Methods S1: Segmentation of the digitizer data**

Circle task

For the circle task a start and end sector were defined as shown in Figure S1. One repetition of the circle task was considered to end and the next one to start when the pen tip moved from the end sector to the start sector. For this study this simple rule was sufficient for unambiguously defining the start and end times of each repetition since none of the participants crossed this border multiple times per circle.

Spiral task

In the spiral task a repetition was considered to start when the pen tip left the start circle and to end when the pen tip entered the end circle (see Figure S1 for illustration). Similar to the circle task this simple rule was enough to unambiguously define the start and end points of each repetition.

Star task

For the star task each individual line-segment drawn by the participant was detected automatically using the following procedure:

1. The distance travelled by the pen-tip was estimated for each data point by computing the cumulative sum of distances between two successive data points.
2. New time series of x and y coordinates in which the data points were equally spaced in distance travelled by the pen were constructed from the original time series, in which the data points are equally spaced in time, using linear interpolation. This transformation removes variation due to varying drawing speed within participants and between participants. The transformation is illustrated in Figure S2.
3. A function which consists of two straight line segments, 25 mm each, joined together at one end was fitted locally to each data point in the x and y time series minimizing the least squares error. This function fits nearly perfectly to the data when the participant is drawing a straight line or an angle (Figure S2).
4. The local minima within 25 mm of the fitting error of this function were searched and the minima in which the angle, which can be computed from the parameters of the functions (line segments) fitted to the *x* and *y* coordinates, was larger than 0.8*pi were classified as points where the participant drew an acute angle (Figure S2).
5. The complete line segments drawn by the participant were found by searching for segments that start by the participant drawing an acute angle in the origin and end by the participant drawing an acute angle in one of the end points of the star, or vice versa.

Segmentation of the ‘elel’ character writing task

To assess micrographia an extensive analysis of the ‘*elel*’ task was performed, employing the pen tip position (x and y coordinates). First, the data was split into separate segments, where each segment represented one line of text, using the *x* coordinate of the pen position. This signal repeatedly shows a slow rise followed by an abrupt descent. The slow rise signifies the writing of ‘elelelel’ and the steep descent indicates that the pen is moved back to the left side of the tablet. Subsequently, the segments corresponding to an ‘e’ or an ‘l’ were identified. The shapes in each line were recognized by using a state vector machine that employs the direction of change of the pen tip position as input.

The data was processed in the following steps.

1. The direction of change (Δ*x*, Δ*y*) of the pen tip position was approximated by dividing the difference between samples that are 10 samples apart by 10. A sample distance of 10 rather than 1 is used as a data smoothing method by filtering irregularities in the input signal. The distance of 10 samples corresponds to a time span of 100 milliseconds, because the signals were sampled at 100 Hz.
2. The signs of Δ*x* and Δ*y* were used to drive a state vector machine. For a ‘perfect signal’ the states cycle through the following states in order (see also Figure S3):

State 1. Δ*x* > 0, Δ*y* > 0: the pen is moving right and up, from the start of the curve toward the rightmost point.

State 2. Δ*x* < 0, Δ*y* > 0: the pen is moving further up but leftward, from the rightmost point to the top.

State 3. Δ*x* < 0, Δ*y* < 0: the pen is moving further left but downward, from the top to the leftmost point.

State 4. Δ*x* > 0, Δ*y* < 0: the pen is moving further down but rightward again, from the leftmost point to the bottom.

Since not all signals were perfect, the actual state vector machine was designed to detect errors and correct for these imperfections. Several additions were implemented.

- During normal operation the state can only change from state N to state N+1 (or from state 4 to state 1). For each of these state changes there is only one component (Δ*x* or Δ*y*) that changes, and that is the only change the algorithm looks for. For example, in state 1 the algorithm only searches for a time point when Δ*x* becomes negative, and then the state goes to state 2.
- A fifth state, state 0, was included which indicates an error or initial state. When in this state, the next state (1,2,3 or 4) is picked based on the signs of Δ*x* and Δ*y* directly (the state vector machine stays in state 0 in case either or both components are 0).
- If the direction component that is not expected to change in a state does change, the state is changed to the error state, and recognition of the current shape is cancelled: the ‘current’ curve is skipped. For instance, this error handling mechanism is evoked if the state is in state 1 (the pen is in the lower right quadrant of the shape, moving right and up) and a downward move is detected.

1. A shape is considered recognized if it went through states 1,2,3,4 and into the next state 1 without errors. The four characteristic points are the four time points where the state changes occurred.

For each recognized segment in the line the rightmost, topmost, leftmost and bottommost points were saved and each of these points was characterized by an *x* coordinate, *y* coordinate and a timestamp. Then the letters were classified as an ‘e’ or an ‘l’ according to the height of the segment. A letter was classified as an ‘e’ when the height of the segment was below the mean letter height and a letter ‘l’ was classified when the height of the segment was above the mean letter height. To finish the analysis, width and height were calculated for each letter.
